# Nanosystems in Cardiovascular Medicine: Advancements, Applications, and Future Perspectives

**DOI:** 10.3390/pharmaceutics15071935

**Published:** 2023-07-12

**Authors:** Hossein Omidian, Niloofar Babanejad, Luigi X. Cubeddu

**Affiliations:** Barry and Judy Silverman College of Pharmacy, Nova Southeastern University, Fort Lauderdale, FL 33328, USA; nb1141@mynsu.nova.edu

**Keywords:** cardiovascular diseases (CVDs), drug-delivery systems, targeted drug delivery, nanoparticles, innovative diagnosis and therapies

## Abstract

Cardiovascular diseases (CVDs) remain a leading cause of morbidity and mortality globally. Despite significant advancements in the development of pharmacological therapies, the challenges of targeted drug delivery to the cardiovascular system persist. Innovative drug-delivery systems have been developed to address these challenges and improve therapeutic outcomes in CVDs. This comprehensive review examines various drug delivery strategies and their efficacy in addressing CVDs. Polymeric nanoparticles, liposomes, microparticles, and dendrimers are among the drug-delivery systems investigated in preclinical and clinical studies. Specific strategies for targeted drug delivery, such as magnetic nanoparticles and porous stent surfaces, are also discussed. This review highlights the potential of innovative drug-delivery systems as effective strategies for the treatment of CVDs.

## 1. Introduction

The field of nanotechnology has emerged as a highly promising area for the treatment of cardiovascular diseases, particularly atherosclerosis and myocardial infarction. Nanosystems possess a range of crucial properties that contribute to their effectiveness in addressing these conditions.

One key property of nanosystems is their ability to enable targeted drug delivery, which leads to enhanced therapeutic efficacy and minimized side effects [1]. These systems can specifically target high-risk atheromatous lesions, inflamed endothelium, and activated cardiac fibroblasts, thereby improving the precision of treatment [2,3,4,5,6]. Additionally, nanosystems facilitate diagnostic imaging by developing molecular imaging agents that specifically target atherosclerotic plaques, thereby enabling improved diagnosis and identification of highly diseased areas [2]. Previous studies have explored the potential of nanoparticles, nanocapsules, and nano-bubbles in targeted detection, investigation, and drug delivery for cardiovascular disorders [5,7].

A significant advantage of nanosystems is their theranostic approach, which combines diagnostic imaging agents with therapeutic molecules. This approach allows for both the diagnosis and localized treatment of atheromatous plaques, resulting in enhanced disease management and improved patient outcomes [2]. Moreover, nanosystems exhibit stimuli-responsive behavior, enabling the controlled release of therapeutic agents in response to internal and external stimuli present in the atherosclerotic environment. This controlled release improves treatment outcomes while reducing systemic side effects [8].

Biomimetic nanoparticles, such as macrophage membrane-coated nanoparticles and platelet membrane-cloaked nanoparticles, mimic the properties of natural cells. These biomimetic properties enhance targeted drug delivery and promote plaque stability [9,10]. Nanosystems, including nanoliposomes and nanoparticle-based drug delivery models, also hold the potential for promoting tissue regeneration and functional recovery in cardiovascular disorders, including myocardial infarction [11,12].

Furthermore, nanosystems enhance the therapeutic potential of various agents, such as nucleic acids, proteins, small-molecule drugs, gas-signaling molecules, and stem cells, for the treatment of cardiovascular diseases [9,13,14]. They also improve drug delivery by providing sustained release and localized therapeutic effects. For instance, injectable hydrogels containing nanocomplexes and therapeutic agents offer improved drug delivery to the targeted site [15,16]. Nanoparticles can overcome biological barriers and enhance targeting to specific tissues or cells, thereby increasing the efficacy of drug delivery while minimizing systemic side effects [16]. Researchers have investigated peptide-modified nanoparticles for targeted delivery to the ischemic myocardium [17].

Nanotechnological approaches enable precise interventions at the molecular and cellular levels, allowing for the early prevention and treatment of cardiovascular diseases [16]. Nanoparticles have demonstrated the potential for noninvasive imaging techniques, monitoring therapy, and tracking disease progression in conditions such as atherosclerosis [18]. Additionally, microparticle and nanoparticle-based therapies that utilize biomaterials encapsulate therapeutic agents, provide sustained release, and improve treatment outcomes [16].

Nanosystems have the potential to improve thrombus treatment by enhancing targeting, prolonging the half-life, and reducing the side effects associated with current thrombolytic drugs [19,20]. Combinatorial therapies using nanoparticles are being investigated, such as polymeric nanoparticles for reducing oxidative stress and muscle damage in cardiac myocytes [21].

In addition, nanosystems were developed to effectively cross the blood–brain barrier (BBB) for stroke treatment [22]. The controlled release of drugs is facilitated by nanosystems such as liposomal amiodarone and platelet-like fusogenic liposomes, which minimize side effects and enhance therapeutic outcomes in cardiovascular diseases [22,23]. These systems also enable the targeted delivery of therapeutic agents to promote angiogenesis, reduce infarction size, and improve cardiac healing [22,24].

Advancements in cell-based drug vehicles offer promising prospects for targeted drug delivery and cell therapy in nanomedicine for cardiovascular applications [24]. Delivery systems utilizing poly(DL-lactide-co-glycolide) (PLGA) and porous silica nanoparticles (pSi) allow the spatiotemporal release of growth factors in cardiovascular applications [25]. Strategies involving albumin-opsonized nanoparticles and nanoplatforms are being explored to overcome the limitations of the blood–brain barrier (BBB) in stroke therapies [26,27].

Furthermore, shear-targeted drug-delivery systems show promise in achieving localized drug release for vascular obstruction sites [28]. Nanoparticles have also been used as carriers for thrombolytic drugs, enhancing thrombolysis and reducing side effects in the management of thrombotic diseases [29]. Nanoprobes have been developed to assess enhanced blood–brain barrier (BBB) permeability, aiming to improve therapeutic outcomes in ischemic stroke [30].

Nanotechnology provides targeted drug-delivery systems for the ablation of abnormal heart muscle cells, management of arrhythmias, and post-operative atrial fibrillation [31,32,33,34,35]. Sirolimus nanoparticles have been investigated for their potential in reducing inflammation response and restenosis rates in lower extremity arteries after balloon injury [35,36]. Poly(lactic-co-glycolic acid) (PLGA)-coated stents loaded with antiproliferative agents enhance endothelialization and reduce restenosis risk after stent deployment [37]. Additionally, drug-loaded nanoparticles show promise in the treatment of severe and life-threatening atherosclerotic lesions in coronary heart disease (CHD) [38]. Mesoporous silica nanoparticles (MSNPs) enhance the therapeutic effects of honokiol in inhibiting neointimal hyperplasia [39].

Nanotechnology also holds potential for intrapulmonary delivery systems in pulmonary hypertension and pulmonary edema. Stable emulsions and nanoparticulate delivery systems are being explored as vehicles for aerosolized pulmonary vasodilators and antihypertensive agents [40].

Nanosystems offer valuable properties for treating cardiovascular diseases, including targeted drug delivery, diagnostic imaging, theranostic approaches, stimuli-responsive behavior, and biomimetic properties. They enhance drug delivery, interventions at the molecular and cellular levels, and imaging capabilities. Nanotechnology shows promise in biomaterial-based therapies, thrombus treatment [19,20], combination therapies, crossing the blood–brain barrier [22], controlled drug release, and cell-based drug vehicles [24]. It has the potential to transform cardiovascular medicine and improve patient outcomes.

This manuscript offers a comprehensive overview of the utilization of nanosystems in the treatment of cardiovascular disorders, encompassing a range of specific conditions such as atherosclerosis, myocardial infarction, thrombosis, ischemia, stroke, arrhythmia, restenosis, hypertension, and pulmonary arterial hypertension. It explores various nanocarriers designed for targeted drug delivery, emphasizing their potential to enhance drug efficacy, overcome biological barriers, and reduce side effects. The manuscript underscores the significance of continued research and development in the field of nanomedicine to drive advancements in cardiovascular therapies and ultimately improve patient outcomes.

## 2. Atherosclerosis

Nano-based systems have been developed for targeted imaging, drug delivery, and therapeutic interventions in atherosclerotic lesions. For instance, nanosystems loaded with superparamagnetic iron oxide nanoparticles enable targeted magnetic resonance imaging of high-risk atheromatous lesions, while also showing potential for drug delivery. Other studies have explored the use of nanoparticles to inhibit restenosis, promote cholesterol efflux, reduce inflammation and oxidative stress, modulate cytokine delivery, and target specific molecules involved in the disease. Nanomedicines offer improved management of cardiovascular diseases by providing targeted drug delivery, imaging, and diagnosis with maximal therapeutic effects and minimal side effects. Additionally, nanotechnology holds promise in enhancing the clinical efficacy of nutraceuticals, developing stimuli-responsive drug-delivery systems, and fabricating biomimetic nanoparticles for atherosclerosis treatment.

One study, [2], introduced a nanosystem that utilized oil-in-water nano-emulsions loaded with superparamagnetic iron oxide nanoparticles. This nanosystem is functionalized with a human antibody (P3) and enables targeted magnetic resonance imaging (MRI) of high-risk atheromatous lesions. It also shows potential for delivering drugs to atheromatous plaques, paving the way for non-invasive imaging and therapeutic interventions in atherosclerosis.

In study [41], researchers investigated a double-layer nano-infusion system’s impact on restenosis in animal models of coronary atherosclerosis. This system combines paclitaxel nanoparticles (PTX-NPs) with vascular endothelial growth factor (VEGF) double-layer nanoparticles (V-P-NPs). The results demonstrated improved vascular endothelial healing and reduced restenosis, highlighting the system’s therapeutic potential in inhibiting vascular restenosis and its clinical significance in coronary artery disease (CAD) treatment.

Study [42] explored the development of liposomes targeting cholesterol crystals, specifically AnxV-Rb1-LPs. These liposomes enhance cholesterol solubilization, reduce accumulation, and promote cholesterol efflux. In vitro studies demonstrate improved anti-inflammatory and anti-apoptotic effects in cholesterol-laden cells. AnxV-Rb1-LPs also effectively accumulate in atherosclerotic plaques, suggesting a potential strategy for cholesterol crystal removal and broader applications in diseases involving excessive cholesterol accumulation.

A novel nanosystem utilizing tetrapod needle-like PdH nanozymes was studied in [43]. These multifunctional nanozymes effectively reduce inflammation and oxidative stress in arterial plaques through ROS scavenging, hydrogen-based anti-inflammatory effects, and autophagy activation. The study highlights the therapeutic promise of engineered nanomedicines in atherosclerosis management and therapy.

Researchers investigated the encapsulation of the anti-inflammatory cytokine interleukin-10 (IL10) into cRGD-conjugated Pluronic-based nanocarriers (NC) [3]. The NC efficiently encapsulated IL10 with high loading efficiency and sustained release capabilities. In vitro and in vivo studies demonstrated improved pharmacokinetics, enhanced accumulation in atherosclerotic lesions, and a significant reduction in plaque size. These findings underscore the potential of nanoparticle-mediated cytokine delivery for inflammation modulation and the prevention of atherosclerosis.

A comparative study [44] delved into the role of targeting ligands in augmenting the efficiency of nanoparticle-based delivery systems for the treatment of atherosclerosis. The study investigated cRGD and collagen IV-targeting peptides conjugated to iron oxide nanoparticles (IONPs) loaded in Pluronic-based nanocarriers. The study revealed the superior performance of cRGD-based targeting in the early stages of atherosclerosis, highlighting its potential for effective plaque detection and treatment.

Lipid-core nanocapsules with docosahexaenoic acid (DHA) as the nucleus and anti-PECAM-1 on their surface were developed for targeted delivery to inflamed endothelium in atherosclerosis [4]. The nanocapsules demonstrated efficient conjugation, a suitable size, and cellular uptake without affecting cell viability. These findings suggest the potential of the developed nanocapsules for pharmaceutical approaches in treating atherosclerosis.

Self-assembled lipid nanoparticles conjugated with a short peptide motif exhibited antagonist effects against CC chemokine receptor 2 (CCR2), a key player in the inflammatory response [5]. Known as CCTV, these nanoparticles demonstrated an affinity to CCR2, inhibited chemotactic migration of monocytes, and downregulated inflammatory responses in macrophages. CCTV enables the targeted imaging of atherosclerosis and shows therapeutic potential by downregulating inflammatory genes and inactivating inflammasomes, holding promise for inflammatory conditions such as cardiovascular disease and cancer.

In [45], a discussion revolved around the emergence of nanomedicines as valuable tools for enhancing the management of cardiovascular diseases, with a particular focus on atherosclerosis. Nanosystems offer targeted drug delivery, imaging, and diagnosis, providing therapeutic benefits. Nanomedicines demonstrate maximal therapeutic effects with minimal side effects, enabling efficient prevention, diagnosis, and treatment of cardiovascular diseases. The surface modifications and multifunctionality of nanomedicines hold considerable potential to improve current treatment modalities.

Nanoparticle-based delivery systems offer the possibility to enhance the clinical efficacy of nutraceuticals in atherosclerosis treatment [1]. By combining nanotechnology and drug-delivery systems, the limitations and adverse effects associated with conventional treatments can be overcome. Further research and experimental evidence are needed to fully explore the benefits of nanomedicine in atherosclerosis treatment and optimize its impact on patient care.

Stimuli-responsive nano-based drug-delivery systems (NDDSs) hold promise in the treatment of atherosclerosis [8]. These NDDSs deliver therapeutic agents to target sites, minimizing adverse effects and enhancing treatment efficacy. The report provides a summary of recent advancements in stimuli-responsive NDDSs for atherosclerosis treatment, covering internal and external stimuli-responsive systems. Various types of organic and inorganic NDDSs were discussed, along with challenges and future prospects in this field.

A comprehensive review [46] emphasized the impact of nanosystems on the treatment of atherosclerosis and restenosis, two major cardiovascular diseases. The study explored common pathogenic factors, discusses therapeutic agents, and highlights high-performance nanodelivery strategies, emphasizing their potential to enhance safety and effectiveness. The review aimed to facilitate the exploration of therapeutic approaches for atherosclerosis and restenosis.

Biomimetic nanoparticle systems for the targeted therapy of atherosclerosis have been investigated [9]. These nanoparticles, coated with a macrophage membrane on rapamycin-loaded PLGA nanoparticles, demonstrate biocompatibility and targeted binding to activated endothelial cells and atherosclerotic lesions. In animal models, the nanoparticle system inhibited atherosclerosis progression and exhibited favorable safety profiles. Biomimetic nanoparticles can serve as safe and effective drug-delivery systems for the treatment of atherosclerosis.

Micro- and nano-bubbles (MNBs) are valuable tools for the detection and treatment of atherosclerosis [7]. MNBs serve as contrast agents for ultrasound imaging and can be modified to target atherosclerotic sites. This review highlighted the fabrication process of MNBs and their applications in bio-nanomedicine, including the diagnosis and remodeling of atherosclerosis, offering potential advancements in disease management.

In [47], the adverse effects of exposure to multi-walled carbon nanotubes (MWCNT) on the progression of atherosclerosis were discussed. Animal models demonstrated that intravenous administration of MWCNTs aggravates atherosclerosis and increases aortic calcification. In vitro studies on human umbilical vascular endothelial cells (HUVECs) revealed that MWCNTs disrupt endothelial tight junctions and induce cell death. These findings highlight the potential risk of MWCNT exposure in contributing to structural and functional changes in the endothelium and the development of atherosclerosis.

Hydrogen sulfide (H_2_S) donors show potential in the treatment of atherosclerosis [48]. This review explored various H_2_S donors with anti-atherosclerotic potential, their transport mechanisms, and design limitations. The use of nano-synthetic technologies in manufacturing H_2_S donors was also discussed, highlighting their potential in creating highly targeted therapies for atherosclerosis.

A biomimetic nanodrug-delivery system (PM@Se/Rb1 NPs) was developed for the targeted treatment of atherosclerosis in [10]. The system utilized platelet membrane coating on selenium (Se) and ginsenoside Rb1 nanoparticles (Se/Rb1 NPs) to enhance plaque stability. The PM@Se/Rb1 NPs exhibited anti-inflammatory and anti-angiogenic effects, effectively delivered drugs to plaques, and show potential synergistic effects when combined with the anticoagulant drug warfarin. This study highlights the potential of biomimetic nanodrugs and their combination with clinical drugs for atherosclerosis treatment.

The impact of plaque morphology on the targeting efficiency of magnetic nanoparticles for drug delivery in atherosclerosis was investigated in [49]. Numerical simulations demonstrated that higher degrees of stenosis reduce particle deposition and adhesive strength on plaques, while longer shoulder lengths increase particle deposition and adhesive strength. Higher degrees of stenosis also resulted in increased shear damage to the plaque. This study emphasizes the importance of considering plaque morphology in optimizing targeted drug delivery for atherosclerosis.

Nanosystems offer promising opportunities in the field of atherosclerosis research and treatment. These innovative approaches, such as targeted imaging, drug delivery, and therapeutic interventions, hold great potential for non-invasive diagnosis and the effective management of atherosclerotic lesions. Nanomedicines, including lipid-based systems [29], nanozymes [43], and biomimetic nanoparticles [9], demonstrate improved outcomes in cholesterol solubilization, inflammation reduction, and plaque stabilization. Furthermore, nanotechnology enables the development of stimuli-responsive drug-delivery systems, enhancing treatment efficacy while minimizing adverse effects. However, careful consideration of factors, such as plaque morphology, and the potential risks associated with certain nanomaterials, such as carbon nanotubes, is essential. Continued research and exploration of nanosystems in atherosclerosis will pave the way for improved patient care and better cardiovascular disease management.

**Figure 1:**
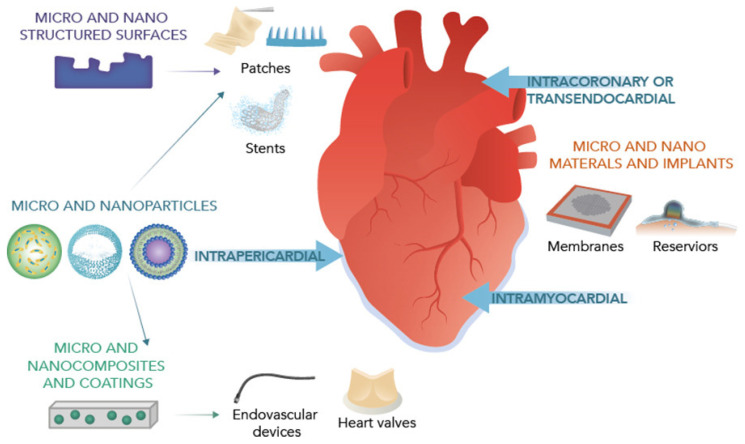
Applications of nanotechnology for treating cardiovascular diseases. Adopted with permission from [16].

The integration of nanotechnology with various approaches, including designer nanoparticles and vascular, implantable, or wearable device technologies, holds promise for the prevention and treatment of cardiovascular diseases (Figure 1) [16].

## 3. Myocardial Infarction

Cardiac remodeling after myocardial infarction (MI) is influenced by the extracellular vesicles (EVs) released by cells. EVs, small lipid bilayer vesicles, contribute to both inflammation and fibrosis in post-infarction cardiac remodeling [13]. Understanding the pathophysiology of this process is essential for developing effective therapeutic strategies.

Nanoliposomes, nano-sized vesicles composed of phospholipid membranes, have emerged as promising drug-delivery systems for cardiovascular disorders. They offer sustained drug release, leading to improved efficacy and targeted delivery to specific areas [50]. Recent advancements in nanoliposomes and nanoparticles as drug delivery vehicles have the potential to transform the treatment of cardiovascular diseases, including atherosclerosis, restenosis, and myocardial infarction.

A novel nanoparticulate system was developed to target the heart after myocardial infarction. This system utilized liposomes conjugated with a ligand specific to the angiotensin II type 1 (AT1) receptor, which is overexpressed in the infarcted heart [11]. In vitro and in vivo studies demonstrated the nanoparticles’ ability to specifically target cardiac cells, offering a potential approach to improve treatment outcomes.

Another innovative approach involves a nanosystem capable of delivering oxygen to the infarcted heart during acute myocardial infarction. Administered via intravenous injection, these nanoparticles specifically target the infarcted area and release oxygen, promoting cardiac cell survival, angiogenesis, and suppressing fibrosis [12]. This nonpharmacological approach offers a potential strategy to rescue the infarcted heart and preserve its function.

The localized delivery of anti-inflammatory drugs to the myocardium using nanosystems has shown promise for treating ischemic heart failure following acute myocardial infarction [51]. Biodegradable polymers, liposomes, hydrogels, and nanoparticles can be utilized for targeted drug delivery. Pre-clinical and clinical studies have demonstrated potential efficacy in preventing post-MI remodeling. However, challenges related to retention, distribution, and dosing need to be addressed for successful clinical application.

Iron oxide nanoparticles (IONPs) enhance the expression of connexin 43 (Cx43), a gap junction protein, in cardiomyoblasts, promoting active gap junctional communication between mesenchymal stem cells (MSCs) and cardiac cells, thereby improving the therapeutic potential of MSCs for myocardial infarction repair [52]. In animal models, modified MSCs with IONPs demonstrated higher cardiac biomarker levels, favorable paracrine profiles, improved animal survival, and enhanced heart function compared to unmodified MSCs. These findings highlight the potential of IONPs in enhancing cell-based therapies.

Furthermore, a nanocomplex combining dendrigraft poly-l-lysine (DGL)-loaded miR-1 inhibitor with low molecular weight heparin (LMWH) has shown promising results for myocardial infarction treatment [53]. Surface modification of the nanocarrier with LMWH helps to overcome microvascular obstruction in the infarct area, facilitating an improved delivery of the miRNA inhibitor. This approach reduces microthrombus formation, enhances miRNA uptake, decreases cardiomyocyte apoptosis, improves cardiac function, and attenuates infarction. 

The field of nanomedicine continues to advance, with promising findings in the treatment and diagnosis of cardiovascular diseases, particularly myocardial infarction (MI). Squalene-loaded gold nanoparticles have emerged as a potential therapeutic alternative for MI due to their small size, unique shape, high encapsulation efficiency, sustained release of cardioprotective effects, low cardiotoxicity, and maintenance of myocardiocyte structural integrity [54]. Impediametric studies indicate their positive impact on impedance, further highlighting their therapeutic potential.

In the realm of targeted drug delivery, a reduced glutathione (GSH)-responsive nanoparticle platform was developed to deliver drugs specifically to activated cardiac fibroblasts (CFs) in the infarct area following MI [6]. This targeted delivery system enhanced the therapeutic efficacy of antifibrotic drugs while minimizing systemic toxicity, offering a promising approach for intervening in cardiac fibrosis.

A nano-platform called PFBT@miR-1-Tat NPs was created to facilitate controlled microRNA delivery and long-term tracking of transplanted mesenchymal stem cells (MSCs) in cardiac regeneration therapy [14]. This platform enables the triggered release of microRNA 1, promoting MSC cardiac differentiation and integration with damaged myocardium. Additionally, the conjugated polymer nanoparticles provide valuable insights into the fate and behavior of therapeutic cells, aiding in the optimization of gene and cell combined therapy for improved clinical translation, thus enhancing stem cell-based cardiac regeneration outcomes.

The local accumulation of exosomes using magnetic nanoparticles shows promise in promoting angiogenesis and improving heart function in infarcted tissue [55]. These nanoparticles, decorated with antibodies that selectively bind to specific markers, can capture and release exosomes in response to a local magnetic field. In myocardial infarction models, this approach leads to reduced infarct size and improved cardiac function. Manipulating endogenous exosome biodistribution through magnetic nanoparticles has the potential to treat various diseases.

**Figure 2:**
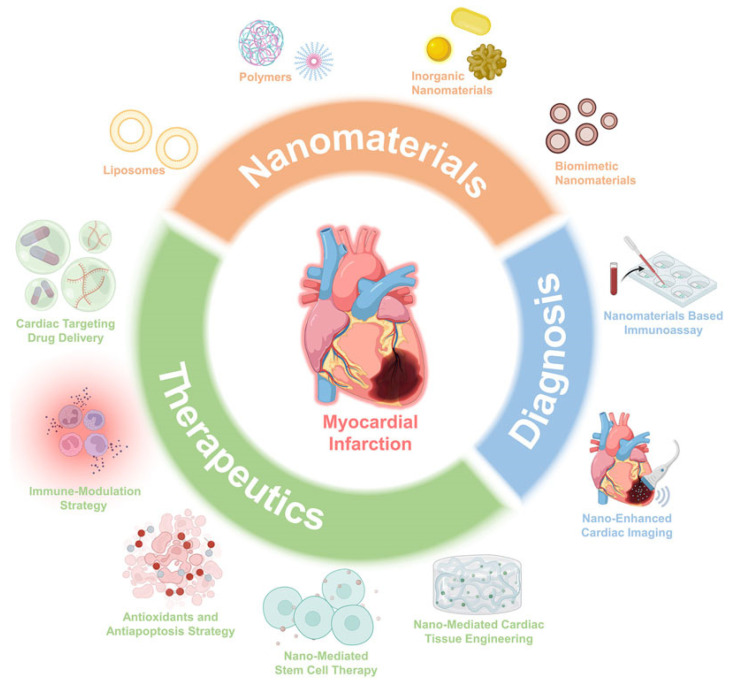
Schematic overview of the advances of nanomaterials and applications for both therapeutics and diagnosis [56].

Nanomaterials offer a range of strategies for the treatment and diagnosis of MI [56]. LV, et al. provided a broad description of nanomaterial applications in both therapeutics and the diagnosis of MI (Figure 2). These materials address the limitations of conventional therapies by targeting the heart, modulating immune responses, providing antioxidants and anti-apoptotic effects, enabling stem-cell therapy, and facilitating cardiac-tissue-engineering. Furthermore, nanomaterials enhance MI diagnosis through immunoassays and improved cardiac imaging. Despite challenges, nanomaterial-based approaches hold immense potential for the next generation of MI treatments, offering hope for improved patient outcomes.

Nano-scale drug-delivery systems (DDS) hold significant promise in enhancing the efficacy and safety of therapeutic agents for cardiovascular diseases [57]. These systems can passively or actively target affected areas, addressing the limitations of conventional treatments. Recent studies have demonstrated the successful targeting of atherosclerosis and myocardial ischemia-reperfusion injury using nano-DDS, with positive effects observed in preclinical animal studies. The clinical development of nano-DDS in cardiovascular medicine holds great potential.

In the realm of diagnostics, nanoscale biosensors combined with nanomaterials offer promising platforms for the rapid, early, reliable, and cost-effective detection of cardiovascular diseases, including acute myocardial infarction [58]. Traditional diagnostic methods using cardiac troponins have limitations, spurring the development of overly sensitive and specific detection methods. Optical and electrochemical biosensors and immunosensors have shown advancements in the detection and quantification of cardiac troponin I and cardiac troponin T, providing valuable tools for improved diagnostic capabilities.

The field of cardiovascular therapy and regeneration is witnessing significant advancements through the utilization of nanotechnology and hydrogel-based approaches. One study focused on the development of an injectable hydrogel containing a nanocomplex of graphene oxide (GO) and the vascular endothelial growth factor-165 (VEGF) gene, which demonstrated improved myocardial capillary density, a reduced scar area, and enhanced cardiac performance in a rat model of acute myocardial infarction [15]. This combined hydrogel-based gene therapy system holds promise for the treatment of ischemic heart diseases.

Hydrogels have emerged as a promising avenue for cardiac tissue regeneration and the recovery of heart function [59]. These versatile materials enable the controlled and minimally invasive delivery of therapeutic cells and proteins. Recent advances in hydrogel-based strategies, including conventional, injectable, smart, and nano- or microgels, were discussed in a comprehensive review. Various biomaterials and encapsulated agents, such as stem cells and proteins, were explored, highlighting the potential of hydrogel-based approaches in advancing drug delivery and cell therapies for cardiac regeneration.

**Figure 3:**
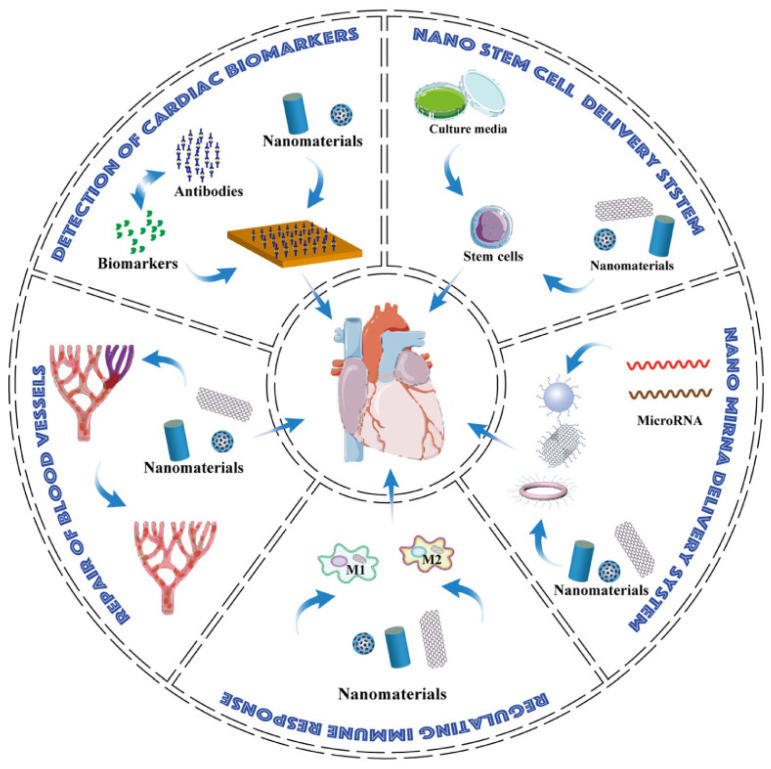
The schematic diagram shows the application of nanomaterials in MI. Nanomaterials help to lower the detection limit of cardiac biomarkers, thus enabling a timely recognition of potential patients. In basic research, Nanomaterials have been explored to promote angiogenesis and regulate immune responses after MI. They also play a promising role in delivering novel biomolecules such as miRNA and stem cells [60].

Nanomaterial-based technologies have shown tremendous potential in improving the prognosis of myocardial infarction (MI) by addressing the disease at the molecular level [60]. This review highlighted the diverse applications of nanomaterials in MI, including precise detection, pro-angiogenesis, immune homeostasis modulation, and miRNA and stem cell delivery (Figure 3). Additionally, promising research areas, such as constructing drug carriers with pro-angiogenetic elements and targeting immune cells, were discussed. Although many strategies are in preclinical stages, they hold immense potential for future clinical applications in MI treatment.

In the pursuit of effective therapies for ischemic heart repair, a study utilized exogenous heparan sulfate proteoglycans (HSPG) as a delivery agent for basic fibroblast growth factor (bFGF) [61]. HSPG demonstrated strong binding with bFGF, enhancing its bioactivity in promoting angiogenesis and protecting it from enzymolysis. The study underscores the potential of extracellular proteins resembling the extracellular matrix to facilitate ischemic heart repair by promoting the activities of heparin-binding growth factors.

Particle-based therapies offer promise in the treatment of cardiovascular diseases such as myocardial infarction (MI) and peripheral artery disease (PAD) [62]. Micro- and nanoparticles are engineered to deliver growth factors, enzymes, and small molecules to the injured tissue, fostering tissue repair and restoring blood flow. The review provides an overview of current particle-based therapies for MI and PAD, highlighting their potential as targeted and sustained therapeutic interventions for these conditions.

Nanotechnology has transformed cardiovascular disease treatment by designing nanoparticles for targeted drug delivery and overcoming biological barriers [16]. These designer nanoparticles can selectively target immune cells, modulate inflammatory processes, and locally deliver therapeutic payloads. The perspective of this study emphasized the potential of nanotechnology in cardiovascular treatment and proposed new areas of research, including vascular devices, nanocomposites, and risk-factor prevention. Nanotechnological approaches have the potential to facilitate the early prevention and treatment of cardiovascular diseases.

The effectiveness of a mitochondria-targeted lipid–polymer hybrid nanosystem loaded with calycosin (CAL) and tanshinone (TAN) was investigated for treating acute myocardial infarction (AMI) [63]. The nanosystem exhibited desirable physicochemical properties and demonstrated high heart accumulation, significantly reducing the infarct size. These findings suggest the potential of the CAL/TAN-loaded nanosystem as an efficient treatment approach for cardiovascular diseases.

In another study, a modified version of vascular endothelial growth factor (VEGF), called IMT-VEGF, was developed for enhanced targeted delivery and therapeutic efficacy in myocardial infarction (MI) [17]. IMT-VEGF demonstrated localization to the ischemic myocardium by interacting with cardiac troponin I (cTnI) when administered intravenously. Treatment with IMT-VEGF resulted in reduced scar size, improved angiogenesis, and enhanced cardiac function in animal models of MI, highlighting the potential of targeted delivery strategies using IMT-VEGF for effective MI therapy.

Nanosystems hold immense potential for transforming the treatment of myocardial infarction (MI). They offer targeted drug delivery, oxygen delivery [12], anti-inflammatory effects [10], and improved stem-cell therapies. Nanoparticles can specifically target cardiac cells, promote angiogenesis, and enhance cell-based therapies. Additionally, nanomaterials enable early and reliable MI detection [60], while hydrogel-based approaches aid in cardiac tissue regeneration. Nanotechnology has the ability to address MI at the molecular level, modulate immune responses, and facilitate precise drug delivery. These advancements pave the way for future clinical applications and improved outcomes in MI treatment.

## 4. Thrombosis

Researchers have extensively investigated nano-based methods to gain a deeper understanding of post-infarction cardiac remodeling and to devise effective therapeutic strategies. Among these methods, nanoliposomes and nanoparticles have emerged as promising systems for delivering drugs in cardiovascular disorders, providing targeted treatment and improved efficacy. The development of innovative nanoparticulate systems has enabled precise targeting of the infarcted heart using ligand-conjugated liposomes. Nanosystems capable of delivering oxygen to the infarcted region during acute myocardial infarction have shown potential in enhancing the survival of cardiac cells and promoting angiogenesis. The localized administration of anti-inflammatory drugs using nanosystems was proven to be effective in preventing post-MI remodeling. Additionally, iron oxide nanoparticles were utilized to enhance the therapeutic abilities of mesenchymal stem cells for repairing myocardial infarction. The combination of miRNA inhibitors with nanoparticles in nanocomplexes has demonstrated promise in the reduction of cardiomyocyte apoptosis and improving cardiac function. Moreover, nanomaterials were employed for the diagnosis of myocardial infarction and the development of biosensors for the early detection of cardiovascular diseases. Hydrogel-based approaches, including nanogels and microgels, have shown potential in the regeneration of cardiac tissue and drug delivery.

In the field of cardiovascular medicine, nanotechnology and biomaterial-based strategies are paving the way for innovative and targeted therapies. One hypothesis focused on utilizing Brij52 niosomal vesicles, a novel nanosystem, for the targeted delivery of antithrombotic agents in cardiovascular diseases [64]. By mimicking platelet activation and leveraging alterations in hemodynamic shear stress, these non-spherical vesicles can selectively target and disrupt thrombi, offering a noninvasive and monitoring-free approach. PEGylation extends the circulation time of these vesicles, while attachment to the von Willebrand factor ensures specific activation at sites of clot formation. This intelligent drug-delivery system shows promise for overcoming the limitations associated with current antithrombotic pharmacotherapy.

To explore targeted drug delivery in arterial thrombosis, von Willebrand factor (VWF)-A1 domain-functionalized nanoparticles (A1-NPs) have been investigated [65]. These nanoparticles exhibit flow-structure dependent adhesion to VWF, with preferential localization at post-stenotic regions in coronary stenosis models. The adhesion of A1-NPs is influenced by coating density and location within the stenosis. This study emphasizes the importance of studying cardiovascular nanomedicines in realistic pathological environments that mimic size, geometry, and hemodynamics. Such engineered nanosystems hold great potential for improving treatment outcomes by facilitating targeted drug delivery in arterial thrombosis.

Monitoring therapy and disease progression in atherosclerosis is a crucial aspect of cardiovascular care. Sodium [(18)F]Fluoride (Na[(18)F]F) has shown promise in tracking plaque progression and assessing the effects of therapeutic interventions in an atherosclerotic mouse model [18]. The uptake of Na[(18)F]F was highest in advanced stages and Warfarin groups, and spotty calcifications observed on CT scans corresponded to dense mineralization. These findings highlight the potential of Na[(18)F]F as a valuable tool for assessing vascular health and evaluating the impact of therapeutic interventions.

Nanoparticles are emerging as powerful tools in the treatment of vascular diseases, transforming traditional approaches. These nano-based delivery systems offer enhanced drug delivery efficiency to the diseased site, overcoming limitations such as systemic exposure, off-target effects, and poor bioavailability [66]. Additionally, theranostic nanoparticles provide the dual functionality of therapeutic and imaging payloads, enabling simultaneous treatment and monitoring. Recent advancements in nanoparticle therapy for atherosclerosis, thrombosis, and restenosis are driving targeted treatment strategies for vascular diseases.

The limitations of current antithrombotic and thrombolytic therapies can be addressed through biomaterial-based strategies. By incorporating nanoscale to macroscale biomaterials, these approaches hold the potential to transform cardiovascular therapies [67]. Formulation methods, controlled release kinetics, and clot-specific drug targeting are discussed in this report, along with the preclinical efficacy of these technologies in cardiovascular applications. Ongoing efforts to develop bioresponsive “smart” biomaterials for precision medicine in cardiology show promising opportunities for advancing the field.

A novel near-infrared II (NIR-II) fluorophore based on rare earth-doped nanoparticles (RENPs@DSPE-mPEG) offers noninvasive mapping and diagnoses of the skeletal system and bone diseases [68]. Imaging at longer wavelengths within the NIR-II window provides higher resolution and signal-to-noise ratio compared to 1064 nm, despite lower quantum yield. RENPs@DSPE-mPEG also demonstrates imaging potential in blood vessels and lymph nodes, while their internalization by white blood cells suggests improved nanoparticle delivery for immunotherapy and cancer-targeted clinical applications.

In the field of cardiovascular medicine, nanotechnology and nanomedicine are transforming the field, offering innovative solutions for targeted drug delivery, diagnostics, and therapy. A multifunctional liposomal system has been developed for the targeted delivery and controlled release of tissue plasminogen activator (tPA), enabling clot dissolution with minimal side effects [69]. These liposomes, PEGylated for stability, are surface coated with cyclic arginine-glycine-aspartic acid (cRGD) to selectively bind to activated platelets. In vitro studies have shown high tPA release upon interaction with activated platelets, resulting in efficient fibrin clot lysis. This system holds promise for enhanced thrombolytic therapy.

Nanomedicine, leveraging the potential of nanotechnology in medical applications, holds great promise in various fields, including cardiovascular diseases and stroke [70]. This review highlights the use of nanomaterials in drug delivery, bio-imaging, and the development of nanoscale devices for diagnosis and therapy. The discussion encompasses FDA-approved nanomaterials, promising clinical applications, limitations, and ongoing developments in the field of nanomedicine. This report highlights the potential of nanomedicine to address unmet medical needs and future challenges.

High-density lipoprotein (HDL)-based nanosystems offer potential for the treatment of cardiovascular diseases. Reconstituted HDL (rHDL) exhibits antiatherothrombotic properties, while HDL nanoparticle platforms enable targeted delivery of therapeutic and diagnostic agents [71]. However, current methods for rHDL synthesis present challenges for large-scale production. To overcome this, a report presents a microfluidics-based manufacturing method for single-step synthesis of HDL-mimicking nanomaterials (muHDL). muHDL demonstrates similar properties to conventionally reconstituted HDL and native HDL, making it a promising platform for medical imaging and drug delivery applications.

A thrombus-targeted nano-fibrinolytic agent has been developed using a magnetofluorescent iron oxide nanoparticle platform conjugated with recombinant tissue plasminogen activator (tPA) [72]. The nanoparticle is derived from an activated factor XIII (FXIIIa)-sensitive peptide to achieve thrombus-targeting capability. In vitro and in vivo studies have demonstrated the superior binding and fibrinolytic activity of this targeted nanoagent compared to control agents. These findings highlight the potential of this nanosystem for effective and safe treatment of thromboembolism, warranting further investigation for clinical applications.

In interventional cardiology, a nanoparticle (NP)-eluting stent system has been developed utilizing cationic electrodeposition coating technology [73]. This NP-eluting stent demonstrates efficient drug delivery, surpassing the capabilities of dip-coated stents in in vitro and in vivo experiments. The nanoparticles are effectively taken up by vascular smooth muscle cells, and in a porcine coronary artery model, fluorescence is observed in the stented segments. The NP-eluting stent holds promise as an innovative platform for less invasive treatment of cardiovascular disease, utilizing nanoparticle-mediated drug delivery.

To address concerns such as restenosis and late stent thrombosis, nanobiotechnological approaches are being explored to improve drug-eluting stents (DESs) [74]. This report provides an overview of these approaches, focusing on the potential of nanotechnology in designing the next generation of stents. It discusses the use of nano-delivery systems to transport drugs and genetic materials, as well as advancements in nanocoatings, nanotubes, and drug delivery for enhanced biocompatibility and vascular healing.

The limitations of current thrombolytic drugs for treating thrombosis can be addressed through targeted nanodrug-delivery systems [19]. Thrombosis is a prevalent cause of cardiovascular diseases, and existing drugs have limitations that hinder their effectiveness. Nanodrug-delivery systems offer potential solutions, including targeted delivery and improved therapeutic efficacy. This report provides an overview of recent advancements in the development of these systems, with a focus on biological and physical responsive approaches to enhance thrombolytic treatment.

**Figure 4:**
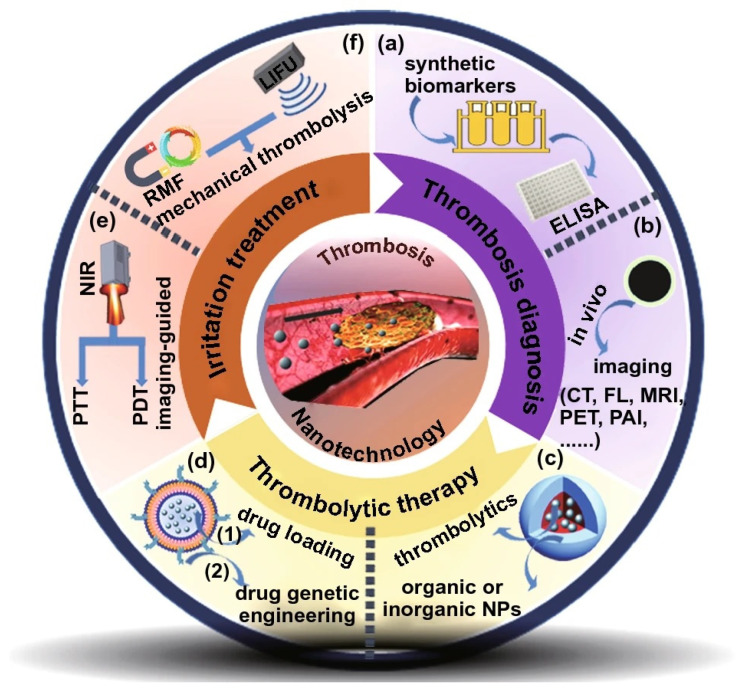
Diagnosis and treatment of thrombosis using nanomedicines [75].

Thrombosis, a significant global health concern with limited diagnostic and therapeutic options, can benefit from advancements in nanosystems. Nanomedicine offers versatile properties for diagnosis, drug delivery, and theranostics, as highlighted in a comprehensive review [75]. Su et al. provided information on successful translation of nanomedicines to the clinical thrombosis treatment using six types of nanoparticle approaches (Figure 4), including in vitro diagnostic kits, in vivo imaging, targeted drug delivery, responsive drug-delivery systems, biological nanostructures, and external irradiation treatments. These nanosystems hold great potential for improving clinical theranostics in thrombosis management. Successful translation of nanomedicines to the clinical thrombosis treatment will enable novel medical diagnostics and therapy to manage thrombosis for personalized medicine.

In the quest for effective arterial thrombosis intervention, a nanoscale arterial thrombosis inhibitor, IQCA-TAVV, was developed [20]. This innovative nanomedicine combines the pharmacophores of anti-platelet agents, a thrombus targeting peptide, and anti-thrombotic nanosystems. In vitro experiments have demonstrated its ability to adhere to activated platelets, attenuate platelet activation, and inhibit platelet aggregation. In vivo studies have shown that IQCA-TAVV effectively targets arterial thrombus and dose-dependently inhibits arterial thrombosis, with remarkable potency surpassing that of aspirin by a factor of 1670. This nanosystem presents promising prospects for arterial thrombosis intervention.

The targeted delivery of thrombolytic agents can be achieved through a unique nanodrug-delivery system known as the urease catalysis micromotor powered neutrophils (NEs) [76]. This system involves the immobilization of urease asymmetrically onto NEs and the loading of urokinase-coupled silver nanoparticles. The resulting UM-NEs Ag-UK system exhibits urease catalytic activity that generates thrust, enabling active targeting of the thrombus. Enriched inflammatory cytokines trigger the release of NEs and concomitant release of Ag-UK, inducing thrombolysis. This innovative system effectively reduces hemorrhagic side effects, promotes thrombolysis, and inhibits rethrombosis, thus offering significant potential for the treatment of thrombotic diseases.

Nanotechnology and biomaterial-based strategies are transforming the field of cardiovascular medicine, particularly in the context of nanosystems and thrombosis. These innovative approaches offer targeted drug delivery, improved diagnostics, and enhanced therapeutic efficacy. By leveraging nanoscale platforms, such as niosomal vesicles [64], nanoparticles [65], and liposomes [69], limitations of current antithrombotic therapies can be overcome. These nanosystems selectively target and disrupt thrombi, provide controlled release of therapeutic agents, and enable simultaneous treatment and monitoring. Additionally, nanotechnology enables the development of bioresponsive biomaterials and nanoscale devices for precision medicine in cardiology. Nanomedicine holds great promise in addressing unmet medical needs and future challenges in the field of cardiovascular diseases and stroke.

## 5. Ischemia/Reperfusion

Ischemia, a condition characterized by inadequate blood supply to tissues or organs, presents significant challenges in the field of medicine. Researchers have actively pursued innovative approaches to address ischemia and enhance patient outcomes. Among these approaches, nanotechnology has emerged as a promising avenue for targeted drug delivery and therapeutic interventions. Nanosystems, consisting of nano-sized particles or structures, offer distinct advantages such as improved drug stability, controlled release kinetics, and the ability to overcome biological barriers. By harnessing nanotechnology, researchers aim to optimize drug delivery, minimize off-target side effects, and enhance treatment effectiveness for various ischemic conditions. This exploration encompasses a wide range of applications, including retinal ischemia-reperfusion injury, cardiac ischemia-reperfusion injury, cerebral ischemic stroke, critical limb ischemia, and ischemic heart failure, among others.

Several studies have investigated the use of nanosystems for targeted drug delivery and therapeutic interventions in various ischemic conditions. One study focused on retinal ischemia-reperfusion injury and examined the intravitreal injection of connexin43 mimetic peptide (MP) encapsulated in poly(lactic-co-glycolic) acid (PLGA) nano- and microparticles (Nps and Mps) [77]. The Nps, with a size of 113 nm, showed efficient drug release, downregulated connexin43, and rescued retinal ganglion cells (RGCs). On the other hand, the Mps, measuring 9 μm, exhibited slower release and delayed effects on connexin43 regulation and RGC preservation.

Another study focused on cardiac ischemia-reperfusion injury and explored the use of multifunctional polymeric nanoparticles for delivering a peptide derived against the alpha-interacting domain (AID) of the L-type Ca(2+) channel [78]. The nanoparticle delivery system demonstrated enhanced perfusion through the myocardium, rapid association with cardiac myocytes, and similar effects on intracellular calcium levels compared to a peptide delivered with a trans-activator of transcription (TAT) sequence. This indicates the potential of nanoparticle-based peptide delivery for attenuating cardiac ischemia-reperfusion injury.

Mitochondrial dysfunction plays a crucial role in cardiovascular disease (CVD) and targeting mitochondria with nanosystems holds promise for therapeutic interventions. Nanotechnology has facilitated the development of mitochondria-targeted drug-delivery systems with improved pharmacokinetics, biocompatibility, and reduced toxicity [79]. These systems target oxidative stress, mitochondrial permeability transition pore opening, and excessive fission, which are potential therapeutic targets for CVD. The review emphasizes the advantages of nanoparticles in targeted mitochondrial therapy compared to non-targeted strategies.

Liposomal drug-delivery systems (DDS) have shown promise in treating cerebral ischemia/reperfusion (I/R) injury by taking advantage of the increased permeability of the blood–brain barrier (BBB) after stroke [80]. However, the entry of liposomes into the brain parenchyma is hindered by early-stage limitations and microvascular dysfunction. To overcome these challenges, researchers have explored leukocyte-mimetic liposomes (LM-Lipo) by incorporating leukocyte membrane proteins onto liposomes. LM-Lipo demonstrated enhanced association with inflamed endothelial cells and the ability to pass through the BBB, highlighting their potential for ischemic stroke treatment.

In the context of cerebral ischemic stroke, cerium oxide nanoparticles encapsulated in poly-(lactide-co-glycolide)-polyethylene glycol copolymer matrices have shown promise as neuroprotective agents [81]. These nanoparticles can cross the blood–brain barrier and function as effective antioxidants by neutralizing free radicals. In experimental models of stroke, the combination of cerium oxide nanoparticles and PEG/PLGA matrices demonstrated significant neuroprotective effects, reducing focal ischemia and brain edema.

Numerous studies have explored the potential of nanosystems for targeted drug delivery and therapeutic interventions in various ischemic conditions. One such study focused on critical limb ischemia (CLI) and developed a nanoparticle-based gene delivery system using a three-arm star block copolymer (3S-PLGA-po-PEG). This system successfully delivered the CYP2J2 gene, which produces epoxyeicosatrienoic acids (EETs) with pro-angiogenic and anti-inflammatory effects. The nanoparticle/pDNA complex exhibited excellent biocompatibility, stability, sustained release, and efficient gene expression. In a hindlimb ischemia model, the complex promoted rapid blood-flow recovery and improved muscle repair compared to naked pDNA. This nanosystem holds great potential for gene-based therapies in CLI and other ischemic conditions [82].

In the treatment of ischemic heart failure (HF), localized delivery of anti-inflammatory drugs to the myocardium using nanosystem-based drug delivery models (NDDM) shows promise [51]. Biodegradable polymers, liposomes, hydrogels, and nanoparticles have been investigated as vehicles for targeted drug delivery. While challenges remain in achieving sufficient retention and distribution, NDDM offer the potential to mitigate off-target side effects associated with systemic administration of anti-inflammatory drugs. Further studies are required to optimize target selection and delivery modalities for successful NDDM-mediated treatment of HF.

Combinatorial therapies for cardiac ischemia-reperfusion injury were explored using multifunctional nanoparticles for simultaneous delivery of the AID peptide and curcumin [21]. The nanosystem exhibited effective attenuation of superoxide production and mitochondrial membrane potential with sustained release rates of curcumin. However, limitations were observed with resveratrol due to a low loading capacity and fast release rates. These findings underscore the importance of drug loading and dissolution in designing effective combinatorial therapies for cardiac ischemia-reperfusion injury.

In the context of ischemic stroke and blood–brain barrier (BBB) crossing, the synthesis of PLGA functionalized magnetic Fe_3_O_4_ nanoparticles (MNP) with L-carnosine peptide (LMNP) composite loaded with dexamethasone demonstrated an efficient drug delivery platform [83]. The nanomaterial exhibited controlled and sustainable drug release kinetics, high drug loading efficiency, satisfactory cytotoxicity, and biocompatibility. The L-carnosine loaded nano-formulation effectively crossed the BBB, highlighting the potential of Fe_3_O_4_ nanoparticles/PLGA polymer as an effective drug carrier for stroke treatment and BBB crossing.

The therapeutic potential of neuropeptide-Y (NPY) in acute myocardial ischemia was assessed using a targeted nanoparticles delivery system [22]. NPY(3-36) was loaded onto copolyoxalate containing vanillyl alcohol (PVAX) using a double emulsification strategy. The PVAX-NPY(3-36) nanosystem demonstrated a significant decrease in infarction size and mortality, improved cardiac function, increased pro-angiogenic factors, and enhanced tissue remodeling compared to the Vehicle group. These results highlight the efficacy of the PVAX-NPY(3-36) nanosystem in promoting angiogenesis and functional improvement in ischemic myocardium.

The field of cardiovascular medicine is witnessing remarkable advancements with the integration of nanoscale drug-delivery systems (DDS) into diagnosis and treatment approaches. These nanosystems offer the potential to passively target pathological sites by leveraging interactions with physiological mechanisms such as vascular permeability. Moreover, active targeting strategies utilize specific structures on the DDS to bind to disease-specific molecules. Studies focusing on atherosclerosis and myocardial ischemia-reperfusion injury have demonstrated promising results in animal models, indicating enhanced efficacy and reduced adverse effects. As a result, the clinical development of nano-DDS holds tremendous promise for transforming cardiovascular medicine [57].

In the realm of myocardial ischemia-reperfusion injury, liposomal amiodarone has emerged as a nano-sized drug-delivery system with significant potential. In an ischemia/reperfusion rat model, liposomal amiodarone exhibited promising outcomes in reducing lethal arrhythmias and hemodynamic changes caused by amiodarone. The liposomes selectively accumulated in the ischemic/reperfused myocardium, enabling targeted drug delivery. Compared to free amiodarone, liposomal amiodarone resulted in a smaller decrease in heart rate and systolic blood pressure. Pre-treatment with liposomal amiodarone also demonstrated a substantial reduction in the duration of lethal arrhythmias and mortality during reperfusion. These findings highlight the potential of nano-sized liposomes as an effective drug-delivery system for cardioprotective agents in the context of ischemia/reperfusion myocardium [23].

Addressing post-myocardial ischemia-reperfusion (MI/R) injury, researchers have developed a platelet-like fusogenic liposome (PLPs) designed to target inflammatory monocytes. This nanosystem delivers miR-21, an anti-inflammatory agent, to the cytoplasm of monocytes through membrane fusion. The reprogrammed macrophages exhibit reparative properties, preserving cardiac function in mice with MI/R. This approach boasts minimal invasiveness and biological safety, positioning it as a promising nanotherapeutic strategy for immunotherapy in the context of cardiac healing post-MI/R injury [24].

Innovative approaches utilizing cell-based drug vehicles have also garnered attention for targeted drug delivery and cell therapy applications. Recent advancements in the functionalization of cells with nano- and microcarriers have shown promise in achieving efficient drug transport. et al. explored various techniques for cell surface modification, carrier internalization, as well as in vivo cell visualization [25].

The application of a nanosystem composed of poly(DL-lactide-co-glycolide) acid (PLGA) and porous silica nanoparticles (pSi) in the delivery of growth factors for tissue engineering has yielded encouraging results. By incorporating vascular endothelial growth factor (VEGF) and platelet-derived growth factor (PDGF) in an electrospun (ES) gelatin patch, spatiotemporal release and localized delivery were achieved. In vitro and in vivo studies have displayed reduced particle internalization, enhanced neovascularization, and upregulation of angiogenesis-related genes. This nanosystem highlights its potential in promoting targeted neovascularization while minimizing cellular toxicity and systemic effects within cardiovascular applications [26].

The integration of nanoscale drug-delivery systems in cardiovascular medicine shows great promise, offering potential advancements in diagnosis, treatment, and therapeutic outcomes. These systems enable targeted delivery, enhanced effectiveness, minimized side effects, and improved tissue repair. As research and development progress, the clinical application of these nanosystems is expected to significantly impact cardiovascular medicine, providing new opportunities for enhanced patient care and outcomes. Nanotechnology has also brought about novel approaches for targeted drug delivery in the management of ischemic conditions and stroke. State-of-the-art research has led to the development of innovative nanosystems, holding significant potential for improving patient outcomes in cerebrovascular and neural injuries.

One such nanosystems utilizes albumin-opsonized nanoparticles co-encapsulated with antioxidases catalase (CAT) and superoxide dismutase 1 (SOD1) to address ischemia-reperfusion (I/R)-induced cerebrovascular and neural injury. This system leverages the use of neutrophils to target inflamed tissues and deliver antioxidases to the brain. By integrating a selenium-containing crosslinker, ferroptosis, a form of cell death, is inhibited. In vitro and in vivo studies have demonstrated the enhanced targeting of neutrophils, efficient penetration of the blood–brain barrier, and a reduction in oxidative damage and apoptosis. This nanosystem shows great potential for treating central nervous system diseases [84].

In the field of ischemic stroke, a bioengineered “nanoplatelet” called tP-NP-rtPA/ZL006e has been developed for the targeted delivery of recombinant tissue plasminogen activator (rtPA) and the neuroprotectant ZL006e. This nanoplatelet consists of a ZL006e-loaded polymeric nanoparticle core enveloped by a platelet membrane shell. It selectively targets the thrombus site and releases rtPA triggered by thrombin. Furthermore, the nanoplatelet can cross the blood–brain barrier to deliver ZL006e to the ischemic brain. Preclinical studies have demonstrated significantly improved anti-ischemic stroke efficacy with tP-NP-rtPA/ZL006e compared to the free drug combination [27].

In summary, nanosystems offer exciting possibilities for targeted drug delivery and therapeutic interventions in various ischemic conditions. Studies have shown the efficacy of nanosystems in retinal ischemia [77], cardiac ischemia-reperfusion injury [78], mitochondrial dysfunction [79], cerebral ischemia-reperfusion injury, ischemic stroke, critical limb ischemia [82], and ischemic heart failure. Nanoparticles, liposomes, polymeric nanoparticles, and cell-based drug vehicles have been explored as delivery systems, demonstrating improved efficacy, reduced side effects, and enhanced tissue repair. These advancements have the potential to transform cardiovascular medicine and improve patient care.

## 6. Stroke

Researchers have made notable progress in leveraging nanotechnology to address stroke, a debilitating neurological disorder. One approach involves the specific recruitment of liposomes into the ischemic brain, taking advantage of the biphasic breakdown of the blood–brain barrier (BBB) during stroke. This enables targeted accumulation of liposomes, leading to neuroprotection and modulation of inflammatory responses. Another promising advancement is the utilization of magnetic nano- and microcarriers loaded with thrombolytic agents, guided by external magnetic fields to the site of vascular occlusion. This allows for targeted and localized release of the therapeutic agent. Moreover, researchers have explored shear-targeted drug delivery, utilizing the physics of stenotic blood vessels to design nanosystems that facilitate localized drug delivery. These innovative approaches showcase the potential of nanotechnology in delivering effective and personalized treatments for stroke patients, addressing the challenges associated with the condition.

In the context of stroke therapy, a mouse model study has demonstrated the selective recruitment of liposomes into the ischemic brain. The study revealed biphasic breakdown of the blood–brain barrier (BBB), allowing targeted accumulation of liposomes during different phases of BBB disruption. This selective brain accumulation coincides with enhanced transcellular transport and impairment of the paracellular barrier. These findings highlight the potential of liposomes in providing neuroprotection and modulating inflammatory responses, offering tailored treatment options for stroke patients [85].

In the pursuit of improving acute ischemic stroke therapy, researchers have developed a magnetic t-PA delivery system. Magnetic nano- and microcarriers loaded with t-PA can be guided to the site of vascular occlusion using external magnetic fields, enabling targeted and focal release of the thrombolytic agent. The feasibility of magnetic drug targeting has been demonstrated in theoretical and preliminary experimental studies using primate models, underscoring the potential of this t-PA delivery system for effective thrombolysis in arteries [86].

Moreover, a review article explores shear-targeted drug delivery as a novel strategy for delivering drugs to sites of vascular obstruction. The article delves into the physics of stenotic blood vessels, emphasizing hemodynamics and transport phenomena. It highlights the development of Shear Activated Nano-particle Aggregates (SA-NPAs) and shear-stress sensitive lenticular liposomes, highlighting their potential for localized drug delivery. In vivo studies have demonstrated the superiority of these technologies compared to conventional treatments. The review also discusses limitations, challenges, and future applications of mechano-sensitive therapeutics in this context [28].

Nanotechnology has emerged as a powerful tool in the field of medicine, particularly in drug-delivery systems for the treatment of ischemic stroke and other neurological diseases. Several studies have demonstrated the significant impact of nanosystems in overcoming barriers and delivering therapeutics to the central nervous system (CNS) effectively. One such nanosystems, leukocyte-mimetic liposomes (LM-Lipo), has shown promise in overcoming inflamed endothelial barriers in ischemic stroke treatment. By incorporating leukocyte membrane proteins onto liposomal membranes, LM-Lipo exhibited enhanced association with inflamed endothelial cells and the ability to penetrate the blood–brain barrier. This innovative approach holds potential for delivering neuroprotective agents and addressing microvascular dysfunction, offering a promising strategy for ischemic stroke therapy through liposomal drug-delivery systems [80].

**Figure 5:**
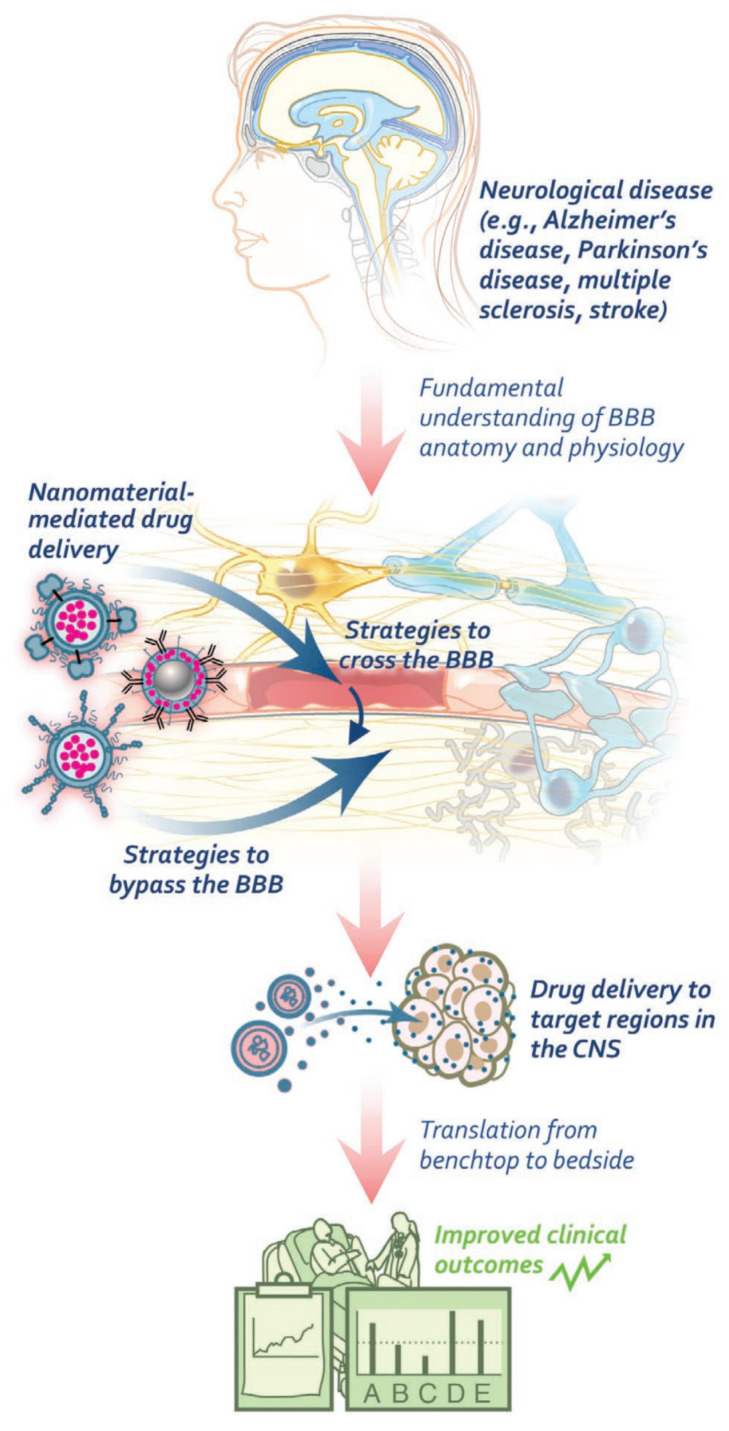
Graphical overview of the process toward nanomaterial-mediated treatment of neurological diseases. Adapted with the permission from [87].

In the quest to overcome the challenges imposed by the blood–brain barrier, nanomaterial-based drug-delivery systems have emerged as a viable solution (Figure 5). A comprehensive review highlights the impact of nanomaterials in improving drug delivery to the CNS. The report explores various mechanisms and strategies for bypassing and crossing the blood–brain barrier using nanomaterials. By providing a comprehensive overview of opportunities and challenges, this review aims to facilitate advancements in nanomaterial-mediated treatment of neurological diseases, contributing to the field of nanomedicine [87].

Another study presents the impact of a novel nanozyme formulation based on PEG-b-poly(aspartate diethyltriamine) (PEG-PAsp(DET)) for chronic dosing. This nanozyme formulation exhibited improved biocompatibility and reduced accumulation in non-target organs. In an ischemic stroke mouse model, it significantly reduced infarct volumes. However, in an ALS mouse model, it did not effectively prevent neuromuscular junction denervation. This study emphasizes the importance of polymer structure in modulating the accumulation of polyion complexes and provides valuable insights for protein delivery using PEG-DET-based nanosystems [88].

The field of nanomedicine has demonstrated its potential in various medical conditions, including cardiovascular diseases and stroke. A comprehensive report highlights the impact of nanosystems in drug delivery and imaging, as well as the development of diagnostic and therapeutic devices. By discussing both FDA-approved nanomaterials and promising candidates, the report sheds light on the pathophysiological basis and potential applications of nanotechnology in medicine. While acknowledging the limitations and challenges, nanomedicine is recognized as a solution to address unmet medical needs and future healthcare challenges [70].

Furthermore, a nanosystem composed of PLGA functionalized magnetic Fe_3_O_4_ nanoparticles (MNP) with L-carnosine peptide (LMNP) composite loaded with dexamethasone (dm@LMNP) has demonstrated its impact in drug delivery for the treatment of ischemic stroke. This nanosystem exhibits controlled and sustainable drug release, satisfactory biocompatibility, and effective blood–brain barrier penetration. With these characteristics, it holds promise as a viable drug carrier for stroke treatment [83].

Nanobiotechnology has emerged as a significant change in the management of cerebral ischemic stroke, offering targeted drug delivery strategies and therapeutic approaches to address the challenges of this neurological disorder. A comprehensive report highlights the significant impact of nanoplatforms, including liposomes, micelles, nanoparticles, and inorganic nanomaterials, in targeted drug delivery. These advancements enable neuroprotection, anti-inflammation, thrombolysis, and blood–brain barrier penetration. By discussing the design and potential of nanobiotechnology, this report provides insights and future perspectives for effective management of ischemic stroke [89].

In the treatment of thrombotic diseases, such as ischemic heart disease and stroke, nano- and microcarriers have shown promising results in thrombolytic drug delivery. Preclinical studies using lipid, polymer, or magnetic nanoparticles loaded with thrombolytic drugs have demonstrated enhanced thrombolysis and reduced side effects. Targeted nanocarriers exhibit increased accumulation in clots, while clinical trials combining lipid-based microbubbles with ultrasound and thrombolytic drugs have shown improved thrombolysis. These findings open new avenues for the development of novel strategies in the management of thrombotic diseases [29].

A bioengineered “nanoplatelet” (tP-NP-rtPA/ZL006e) has been developed for targeted delivery of thrombolytic and neuroprotectant drugs in ischemic stroke treatment. This innovative nanoplatelet comprises a platelet membrane shell conjugated with thrombin-cleavable rtPA and a ZL006e-loaded nanoparticle core. By selectively targeting the thrombus site and releasing rtPA upon thrombin activation, tP-NP-rtPA/ZL006e exhibits superior efficacy in preclinical tests. It reduces the ischemic area and reactive oxygen species levels compared to the free drug combination, highlighting its potential as a promising treatment option for ischemic stroke [27].

In the context of ischemic stroke, the use of thrombolytic agents is limited by a short therapeutic window and the risk of brain hemorrhage. Nano-neuroprotectants offer a promising solution by targeting ischemic regions and crossing the disrupted blood–brain barrier. A study demonstrates that nanoprobe uptake in ischemic regions depends on nanoparticle diameter, with a determined upper limit of pore size in ischemic vasculature. These findings underscore the potential of nano-neuroprotectants in leveraging locally enhanced blood–brain barrier permeability for targeted therapy, opening new avenues for precise and effective treatment of ischemic stroke [30].

Nanosystems hold tremendous promise in the field of stroke therapy, offering targeted drug delivery and neuroprotective strategies. Studies have demonstrated the potential of liposomes, magnetic delivery systems, and shear-targeted drug delivery [28] in overcoming barriers and delivering therapeutics to the brain effectively. Nanosystems, such as leukocyte-mimetic liposomes [80] and nanomaterial-based drug-delivery systems, show great potential in addressing microvascular dysfunction and improving drug delivery to the central nervous system. Additionally, innovative nanosystems, including nanozyme formulations and bioengineered nanoplates, exhibit promising results in reducing infarct volumes and enhancing thrombolysis. These advancements in nanomedicine offer hope for improved treatments and better outcomes for stroke patients. The field of nanobiotechnology further enhances targeted drug delivery, providing neuroprotection, anti-inflammation, and blood–brain barrier penetration. Nanosystems offer a solution to the challenges posed by ischemic stroke, transforming the management of this neurological disorder and paving the way for future advancements in stroke therapy.

## 7. Arrhythmia

Researchers have made significant strides in utilizing nanotechnology to address arrhythmia, a prevalent cardiac disorder characterized by irregular heart rhythms. One approach involves the development of cardiac-targeting nanoparticles loaded with therapeutic agents, enabling precise delivery to heart muscle cells. In preclinical studies, these engineered nanoparticles have demonstrated targeted ablation of myocytes while sparing damage to other cell types. By enhancing ablative technologies, this nanosystem holds promise in reducing complications associated with non-specific energy delivery and improving patient outcomes. Nanosystems have also transformed the targeted delivery of antiarrhythmic drugs, providing enhanced drug delivery and therapeutic effects. Additionally, nanolipid-based delivery systems and nanostructured films loaded with therapeutic agents have shown potential in suppressing inflammation, preventing post-operative complications, and alleviating arrhythmia.

Moving to the field of arrhythmia, a novel nanosystem utilizing cardiac-targeting nanoparticles loaded with a photosensitizer shows promise in the specific delivery of therapeutic agents to heart muscle cells, or myocytes. In preclinical studies, these engineered nanoparticles achieved targeted ablation of myocytes without causing damage to other cell types in the heart. By enhancing ablative technologies for the treatment of cardiac arrhythmias, this nanosystem holds the potential to reduce complications associated with nonspecific energy delivery and improve patient outcomes [31].

Nanosystems have transformed the targeted delivery of antiarrhythmic drugs, offering improved drug delivery and therapeutic outcomes. A comprehensive article discusses various nanocarriers, including lipid nanocapsules, liposomes, niosomes, solid lipid nanoparticles, and polymeric nanoparticles, highlighting their effectiveness in delivering drugs to specific tissues. The potential of nanotechnology in enhancing drug delivery is emphasized, along with examples of nanodrug-delivery systems encapsulating antiarrhythmic agents [32].

In a study focused on suppressing perioperative inflammation and post-operative atrial fibrillation, a nanostructured film loaded with dexamethasone (DEX) and amiodarone (AMIO) demonstrated significant impact. The film, composed of Parylene-C (PPX), was applied epicardially in a rabbit model, resulting in reduced adhesions, decreased fibrosis, and diminished atrial fibrillation duration. These findings suggest the potential of DEX/AMIO-loaded PPX films as a promising strategy for preventing post-operative cardiac complications [33].

Liposomal amiodarone, a nano-sized drug-delivery system, was investigated for its impact on lethal arrhythmias and hemodynamic parameters in an ischemia/reperfusion rat model. The liposomes, designed for selective delivery of amiodarone to ischemic/reperfused myocardium, demonstrated reduced mortality due to arrhythmias and minimized negative hemodynamic changes compared to free amiodarone. These findings highlight the potential of nano-sized liposomes as a promising drug-delivery system for targeted delivery of cardioprotective agents to ischemic/reperfused myocardium [23].

Atrial fibrillation (AF), a common and debilitating cardiac arrhythmia, can benefit from the use of nanodrug-delivery systems. These systems offer improved therapeutic outcomes, reduced recurrence rates, and enhanced safety compared to traditional drug therapy and ablation. The targeted delivery capabilities of nanosystems hold promise for more effective and economical treatment strategies, addressing the clinical challenges associated with AF management and improving patient outcomes [34].

A nanolipid-based delivery system for Guanfu base A (GFA) shows promising potential in the treatment of arrhythmia. Composed of Poloxamer 188, lecithin, and medium-chain fatty acid, this system enhances the targeting and absorption of GFA, leading to improved clinical outcomes. The nanolipids exhibit excellent stability, safety, and biocompatibility, effectively alleviating arrhythmia in animal models, specifically ventricular ectopia and ventricular tachycardia. Furthermore, the nanosystem demonstrates longer circulation time and enhanced heart specificity, positioning it as an ideal carrier for GFA in cardiovascular disease treatment [35].

## 8. Restenosis

Researchers have made notable progress in utilizing nanotechnology to address restenosis, a condition characterized by the re-narrowing of blood vessels following interventions such as angioplasty or stent placement. One approach involves the development of nanoliposomal formulations loaded with therapeutic agents, such as sirolimus, for targeted delivery. Studies have demonstrated that the adventitial delivery of Nanolimus, a sirolimus nanoliposomal formulation, effectively reduces neointima area and luminal stenosis in lower extremity arteries, indicating its potential in mitigating vascular restenosis. Double-layer nanoparticles have also shown promise in inhibiting restenosis and promoting vascular healing in animal models of coronary atherosclerosis. Nanoparticle therapy, encompassing various nanosystems, such as liposomes and nanoparticles, has emerged as a valuable approach for the targeted treatment of vascular diseases, offering enhanced drug-delivery capabilities and potential theragnostic applications. Furthermore, nanoparticle-eluting stents and nanoparticle-coated surfaces have demonstrated positive outcomes, including reduced restenosis, improved endothelial healing, and inhibited smooth muscle-cell growth.

In a study focused on lower extremity arteries, a sirolimus nanoliposomal formulation called Nanolimus was delivered through an infusion catheter. The in vitro characterization revealed the long-term stability of the nanoliposomes and sustained drug release. In vivo experiments using swine models demonstrated a significant reduction in the neointima area and luminal stenosis in arteries treated with Nanolimus, indicating the effectiveness of adventitial delivery in mitigating vascular restenosis [36].

Another study investigated the impact of double-layer nano-infusion therapy on restenosis in animal models of coronary atherosclerosis. The double-layer nanoparticles exhibited good characteristics and high drug-encapsulation efficiency. The observation group treated with these nanoparticles showed improved vascular endothelial healing and reduced occurrences of vascular restenosis compared to the control group, suggesting the potential clinical value of this therapy in inhibiting restenosis and promoting vascular healing [41].

One report provided an overview of recent advancements in nanoparticle therapy for vascular diseases, including atherosclerosis, thrombosis, and restenosis. Nanoparticles offer enhanced drug delivery capabilities and the potential for theranostic applications, combining therapy and imaging. These nanosystems have a significant impact on targeted treatment strategies for vascular diseases [66].

Another study assessed the impact of sirolimus nanoparticles delivered through a porous angioplasty balloon in porcine arteries. The sirolimus concentrations remained therapeutic for an extended period, with higher levels observed in coronary artery sites. Sirolimus nanoparticle treatment resulted in reduced stenosis compared to balloon angioplasty alone, demonstrating the feasibility and efficacy of local sirolimus nanoparticle delivery for long-term intra-arterial therapy [90].

Furthermore, a study investigated the impact of a femtosecond laser surface treatment on a drug-loaded PLGA coating to enhance endothelialization and reduce restenosis risk after stent deployment. Different surface patterns were created using femtosecond laser energies, influencing the drug-loading capacity and release profile. The rapamycin-eluting stent, with a specific surface pattern, showed promising potential for endothelialization and resistance to restenosis, offering a novel approach for vascular plaque management [37].

Non-invasive strategies utilizing advanced drug-delivery tools, such as liposomes, micelles, and nanoparticles, have emerged as promising approaches for the treatment of coronary heart disease (CHD). These innovative approaches have demonstrated improvements in atherosclerotic lesions and reduced restenosis rates following stenting. Clinical trials investigating the nanoparticle formulations of drugs such as paclitaxel and alendronate have shown promising safety profiles in patients undergoing percutaneous coronary intervention. The use of non-invasive drug-delivery systems holds significant potential for effectively treating CHD and addressing severe and life-threatening lesions [38].

In a study focused on stent technology, a nanoparticle (NP)-eluting stent system was developed using a cationic electrodeposition coating technology. The NP-mediated drug-delivery system exhibited efficient uptake by the vascular smooth muscle cells in vitro and demonstrated sustained drug release in a porcine coronary artery model in vivo. The NP-eluting stent exhibited comparable performance to bare metal stents, showing minimal injury, inflammation, improved endothelial recovery, and reduced neointima formation. These findings highlight the potential of NP-eluting stents as an innovative and less invasive platform for targeted drug delivery in cardiovascular disease treatment [73].

Nanotechnology and biotechnology are driving advancements in the field of drug-eluting stents (DESs). This report provided an overview of nanobiotechnological strategies for biomedical implants, with a particular focus on next-generation stent design. Various nano-delivery systems, including those for drug, gene, and oligonucleotide transport, were discussed for promoting vascular remodeling. The review emphasizes the importance of nanocoatings, nanotubes, and drug-delivery approaches to enhance biocompatibility, inhibit smooth muscle-cell growth, and promote endothelial cell proliferation in DESs [74].

Furthermore, this report highlights the impact of nanosystems in the treatment of atherosclerosis and restenosis, which are major contributors to cardiovascular diseases (CVDs). The shared pathogenic factors of these conditions have led to the development of co-target treatments. The review discussed various therapeutic agents, including nucleic acids, proteins, small-molecule drugs, and gas-signaling molecules. Nanodelivery strategies play a crucial role in improving the safety and effectiveness of these agents through systemic and local administration. The report provides an overview of emerging therapeutic molecules and nanodelivery strategies, aiming to inspire further exploration in the treatment of these diseases [46].

In another study, a nanosystem utilizing mesoporous silica nanoparticles (MSNPs) loaded with honokiol, a polyphenol with potential therapeutic effects, was investigated for preventing vascular restenosis. The honokiol-loaded MSNPs demonstrated inhibitory effects on the proliferation and migration of vascular smooth muscle cells (VSMCs) and effectively suppressed intimal thickening in a rat model of balloon injury. This nanosystem presents a promising strategy for preventing restenosis and enhancing the therapeutic potential of honokiol in the context of vascular interventions [39].

This report focused on the significant impact of nanosystems in localized drug delivery from drug-eluting stents (DESs), specifically highlighting a nanosystem with a micro- to nanoscale network assembly. By incorporating copper ions, epigallocatechin gallate (EGCG), rapamycin, and bivalirudin (BVLD), this nanosystem demonstrated the sustained release of rapamycin and nitric oxide (NO), promoting endothelial cell growth while inhibiting smooth muscle-cell proliferation. The coating exhibited long-term antithrombotic efficacy, improved endothelial regeneration, and reduced restenosis. This polyphenol-network-mediated surface chemistry offers a promising approach for multifunctional surface engineering in DESs, advancing their therapeutic capabilities [91].

Studies have shown that nanosystems, such as sirolimus nanoliposomes and double-layer nanoparticles, can effectively reduce neointima formation and promote vascular healing [41]. Nanoparticle therapy offers enhanced drug-delivery capabilities and theranostic applications for targeted treatment strategies. Additionally, innovative approaches utilizing drug-loaded coatings and femtosecond laser surface treatments [37] show potential for reducing restenosis risk and enhancing endothelialization. Non-invasive drug-delivery systems, including liposomes and nanoparticles, have demonstrated improvements in atherosclerotic lesions and restenosis rates following stenting. Nanoparticle-eluting stents and nanocoatings provide less-invasive platforms for targeted drug delivery, exhibiting improved biocompatibility, reduced neointima formation, and enhanced endothelial recovery. Nanosystems loaded with therapeutic agents, such as honokiol and polyphenols, show inhibitory effects on smooth muscle-cell proliferation and intimal thickening, presenting promising strategies for preventing restenosis.

## 9. Hypertension/Pulmonary Arterial Hypertension

Nanotechnology has demonstrated potential in addressing hypertension, a common cardiovascular condition. Researchers have made advancements in developing innovative nanosystems for targeted delivery of antihypertensive agents. These nanosystems enhance drug solubility, enable controlled release, and hold promise for improving therapeutic outcomes. These developments provide opportunities for more effective and personalized interventions in the management of hypertension.

In the field of hypertension, a novel nanosystem based on a stable water-in-perfluorooctyl bromide (PFOB) emulsion was developed for intrapulmonary delivery of pulmonary vasodilators. The nanosystem was evaluated for its efficacy in reversing hypoxic pulmonary vasoconstriction (HPV) during acute hypoxia. The results demonstrated that aerosolized delivery of the vasodilators through the nanosystem significantly reduced pulmonary artery pressure compared to control groups. This approach, using pressurized, metered dose inhalers (pMDI), holds promise for the treatment of pulmonary vascular pathologies, providing a targeted and effective therapy for managing hypertension-related conditions [40].

Furthermore, a nanoparticulate-delivery system utilizing Eudragit RS100 was developed to improve the solubility and bioavailability of nebivolol, a beta-blocker used for hypertension and cardiovascular diseases. Through an optimization of formulation and process variables, the nanoparticles exhibited a smooth and spherical morphology with a nano-sized particle size. The in vitro drug release study demonstrated prolonged release and reduced burst release compared to the pure drug powder. This nanosystem has the potential to enhance the therapeutic effectiveness of nebivolol by improving its solubility and controlled-release profile, leading to optimized hypertension treatment outcomes [92].

In a study investigating pulmonary arterial hypertension (PAH), the efficacy of ONO1301 nanospheres (ONONS) was evaluated in a rat model. The targeted delivery of ONONS resulted in increased levels of hepatocyte growth factor (HGF)-expressing fibroblasts and HGF expression, while reducing inflammatory cytokines. A histological assessment showed improvements in pulmonary vasculature thickness and decreased smooth muscle-cell proliferation. Additionally, the right ventricle pressure/left ventricle pressure was improved in the ONONS group. These findings demonstrate the effectiveness of the selective delivery of ONONS to damaged lungs in ameliorating PAH, highlighting its potential as a targeted therapy for managing this condition [93].

In the realm of hypertension, nanosystems have shown significant potential in enhancing drug delivery and improving therapeutic outcomes. One such system is the controlled-release system FA-VP5-LNPs, developed for the oral delivery of the antihypertensive peptide VP5. This system exhibited high stability, controlled-release behavior, and improved cellular uptake and intestinal absorption. In vivo bioavailability studies demonstrated significantly higher absorption of FA-VP5-LNPs compared to free VP5. Furthermore, FA-VP5-LNPs maintained a strong antihypertensive effect for six days, suggesting the potential to reduce administration frequency and improve patient compliance. Importantly, these nano-formulations demonstrated no toxicity, highlighting their ability to enhance peptide delivery and improve antihypertensive effects [94].

Nanomedicine offers a promising approach for the controlled and targeted release of antihypertensive drugs, overcoming the limitations of conventional therapies. Biopolymer-based nanocarrier systems, particularly chitosan, have shown potential in enhancing oral bioavailability, reducing hydrophobicity, and prolonging the plasma half-life of antihypertensive drugs. This review emphasized the mechanisms of antihypertensive drugs, the drawbacks of current strategies, and the advantages of nanomedicine in this context. Recent reports of bio-based nano/micro-carriers with different antihypertensive drugs, focusing on chitosan as a carrier, were analyzed, highlighting its unique properties and potential in improving the efficacy of antihypertensive therapies [95].

Ciliopathies, characterized by abnormal primary cilia function, were investigated in terms of their response to nanosystems. Two cilia-targeted nanoparticle drug-delivery systems (CTNDDS), CT-DAu-NPs, and CT-PLGA-NPs, were developed and compared. These systems efficiently targeted primary cilia and outperformed the conventional drug fenoldopam in murine models by eliminating side effects. While both CTNDDS showed similar therapeutic effects, CT-PLGA-NPs exhibited a tendency to restore ciliopathy parameters closer to normal levels. These findings underscore the potential of nanomaterial-based personalized treatments for ciliopathies without the need for new drugs, offering hope for more effective and tailored interventions [96].

## 10. Concluding Remarks

There are certain challenges that need to be addressed for the successful clinical implementation of nanosystems in the treatment of cardiovascular disorders. In the context of ischemic heart failure following acute myocardial infarction, one of the key challenges lies in achieving optimal retention, uniform distribution, and appropriate dosing in targeted drug delivery. These factors play a crucial role in maximizing the therapeutic effects of nanoparticles in the myocardium. It is also important to carefully consider plaque morphology and the potential risks associated with specific nanomaterials, such as carbon nanotubes, in order to ensure patient safety and minimize any potential adverse effects.

In the field of stroke therapy, there are challenges in effectively delivering drugs to the brain. Although liposomes, magnetic delivery systems, and shear-targeted drug delivery show promise, further research is required to optimize these approaches and ensure the efficient delivery of therapeutics to the central nervous system. Overcoming microvascular dysfunction and improving penetration through the blood–brain barrier are ongoing challenges that need to be addressed in order to enhance the efficacy of nanosystems in stroke treatment.

In the context of arrhythmia, achieving specific and targeted delivery to heart muscle cells while avoiding harm to other cell types presents a challenge. Nanosystems offer potential solutions by enabling the use of cardiac-targeting nanoparticles loaded with therapeutic agents. However, further research is necessary to refine these delivery systems and enhance their precision and effectiveness in treating arrhythmias.

When it comes to restenosis and vascular diseases, optimizing therapeutic efficacy and minimizing adverse outcomes are key challenges. Nanosystems show promise in reducing neointima formation and promoting vascular healing, but further research is needed to improve their drug-delivery capabilities and expand their theragnostic applications. Ensuring the biocompatibility of nanoparticle-eluting stents and nanocoatings, as well as developing effective preventive strategies for restenosis, are areas that require additional investigation.

While nanosystems offer significant advantages in targeted drug delivery, diagnostics, and therapy for cardiovascular disorders, it is important to carefully consider and address these challenges for their successful clinical implementation. Continued research and exploration of nanosystems in cardiovascular medicine will be crucial in overcoming these challenges and realizing their full potential in improving patient care and outcomes.

Disclosure: The authors partly used OpenAI’s large-scale language-generation model. The authors reviewed, revised, and edited the document for accuracy and take full responsibility for the content of this publication.

## Data Availability

Not applicable.
